# Impact of Right Atrial Appendage Ligation *vs.* Repair on Serum Atrial Natriuretic Peptide, Brain Natriuretic Peptide, and Atrial Fibrillation following Coronary Artery Bypass Grafting

**DOI:** 10.21470/1678-9741-2021-0574

**Published:** 2025-08-22

**Authors:** Murat Fatih Can, Hüseyin Sicim, İsmail Selçuk, Ümmühan Nehir Selçuk, Veysel Temizkan

**Affiliations:** 1 Department of Cardiovascular Surgery, Adana Çukurova State Hospital, Adana, Türkiye; 2 Department of Cardiovascular Surgery, Kırklareli Training and Research Hospital, Kırklareli, Türkiye; 3 Department of Cardiovascular Surgery, Sultan Abdülhamid Han Training and Research Hospital, İstanbul, Türkiye; 4 Department of Cardiovascular Surgery, Dr. Siyami Ersek Thoracic and Cardiovascular Training and Research Hospital, İstanbul, Türkiye

**Keywords:** Atrial Natriuretic Factor, Coronary Artery Bypass, Atrial Fibrillation.

## Abstract

**Objective:**

In this study, we aimed to compare the levels of serum atrial natriuretic peptide (ANP) and brain natriuretic peptide (BNP) with ligation and primary repair of right atrial appendage after venous decannulation procedure in isolated coronary artery bypass grafting (CABG) and their relationship with postoperative atrial fibrillation (POAF).

**Methods:**

In this prospective randomized study, 38 patients who underwent isolated CABG in Haydarpasa Training Hospital between March 2015 and November 2015 were included. Patients were divided into two groups whose atrial appendage were ligated (group A) or primary repaired (group B) after right atrial appendage decannulation. Both groups were evaluated in terms of perioperative serum ANP/BNP levels and POAF incidence. ANP/BNP levels were measured by taking blood samples through the central venous catheter on the preoperative day and postoperative days 1 and 3.

**Results:**

While six POAF incidents were observed in group A, there were none in group B. There was no statistical difference between the groups (P > 0.05) in the evaluation of ANP/BNP levels. POAF rate in group A was statistically significantly higher than in group B (P < 0.05).

**Conclusion:**

No significant difference in perioperative ANP/BNP levels was observed between the two groups. Also, no correlation between ANP/BNP levels and POAF were detected. Development of POAF significantly increased in group A. Therefore, we advocate that the prevalence of atrial fibrillation might be reduced in patients who had undergone right atrial repair with primary repair method.

## INTRODUCTION

**Table:** 

Abbreviations, Acronyms & Symbols				
AF	= Atrial fibrillation		DM	= Diabetes mellitus
ANP	= Atrial natriuretic peptide		ECG	= Electrocardiogram
BMI	= Body mass index		EF	= Ejection fraction
BNP	= Brain natriuretic peptide		HT	= Hypertension
CABG	= Coronary artery bypass grafting		PAD	= Peripheral arterial disease
CCT	= Cross-clamping time		POAF	= Postoperative atrial fibrillation
CKD	= Chronic kidney disease		RA	= Right atrial
COPD	= Chronic obstructive pulmonary disease		SD	= Standard deviation
CRF	= Chronic renal failure		TBT	= Total bypass time

The number of cardiac operations is increasing day by day in parallel with the increase in patient population. Therefore, it is becoming more important to prevent postoperative morbidity and mortality. The most common complications after isolated coronary artery bypass grafting (CABG) are arrhythmias. Among these, postoperative atrial fibrillation (POAF) is the most common. Its incidence in isolated CABG patients varies between 20 and 40%^[1]^. Although it is generally considered as a temporary and harmless complication, it is a problem associated with increased early and late mortality^[1,2]^. It is especially important because of the increase in risk of thromboembolism, decrease in contractile functions of the heart, and increase in length of hospital stay and costs.

Despite many studies, its etiology has not been fully elucidated yet. Advanced age is seen as the most important risk factor for POAF. Besides, not using preoperative beta-blockers, prolonged intraoperative cross-clamping time (CCT), localization of venous cannulation, postoperative pneumonia, prolonged ventilation, and electrolyte disturbances are considered as risk factors^[3-5]^.

Myocyte granules in the right atrial (RA) appendage are the region where atrial natriuretic peptide (ANP) secretion is the highest and brain natriuretic peptide (BNP) secretion is partial. It has been demonstrated in many studies that there is an increase in ANP and BNP levels in atrial fibrillation (AF)^[6]^. Although in the current literature ANP and BNP levels were determined by preserving the RA appendage anatomy in isolated coronary bypass operations, we could not find any study comparing them with POAF. In this study, we investigated the effect of ligation applied to the RA appendage after decannulation and primary repair method on serum ANP/BNP levels and their relationship with POAF in coronary artery bypass operations.

## METHODS

This study was approved by the ethics committee of Istanbul Haydarpaşa Numune Training and Research Hospital (HNEAH-KAEK 2015/14) and it was performed prospectively in patients who underwent isolated CABG between March 2015 and November 2015 in the Cardiovascular Surgery Service of Gülhane Military Medical Academy Haydarpaşa Training Hospital. Patients older than 18 years of age who had a coronary bypass decision after coronary angiography and accepted to have surgery were included in the study. A total of 38 patients included in the study were randomly divided into two groups (group A, ligation, and group B, primary repair). Those with moderate and severe valvular dysfunction on echocardiography, ejection fraction (EF) < 50%, pulmonary pressure > 30 mmHg, chronic renal failure (CRF), previous cardiac surgery, and 18 younger patients were not included in the study.

A total of 5 cc blood samples were taken from the central venous vascular access into a routine tube with red cap, at the same time, from the patients on the preoperative day and postoperative 1^st^ and 3^rd^ days. The samples were centrifuged at 5000 rpm for five minutes at 4°C (Hettich Universal 320, Buckinghamshire, England) in the biochemistry laboratory as soon as possible after collection. The serum portion was separated and taken as 1 ml with pipettes, placed in Eppendorf Tubes®, and stored in a freezer at -80°C (Innova Brunswick Scientific Ultra Low Temperature Freezer, United States of America). At the end of the study, the samples were dissolved at room temperature and serum ANP (Human Atrial Natriuretic Peptide (ANP) ELISA Kit, SunRed Biotechnology Company, Shanghai) and BNP (Human Brain Natriuretic Peptide (BNP) ELISA Kit, SunRed Biotechnology Company, Shanghai) kits were used in the biochemistry laboratory (BioTek ELx800 microplate reader). CA-200 washer (USA) and CIOM devices (China) were used.

### Surgical Technique

**Fig. 1:**
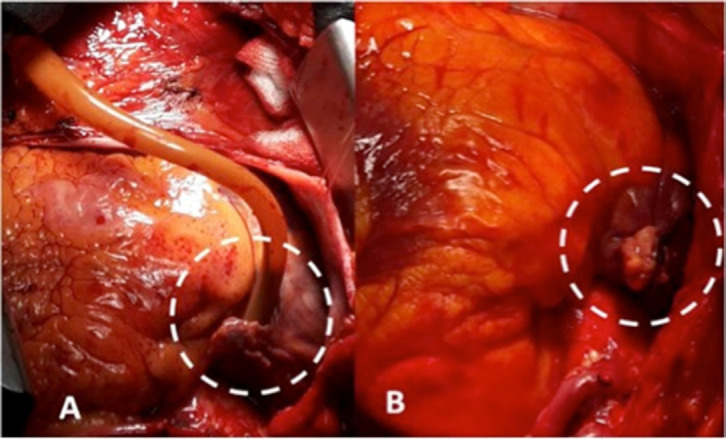
A) Squeezing the right atrial appendage with the help of snare after venous decannulation. B) Ligation of the right atrial appendage.

In the ligation group (group A), aortic purse and venous purse sutures were placed with 2/0 Ethibond sutures at the stage of entering the heart-lung machine. After the aortic cannula was placed, the two-stage venous cannula was inserted into the RA appendage and tightened with the help of purse suture snare. After venous decannulation at the stage of separation from the heart-lung machine, the RA appendage was tied with 1/0 silk by tightening the snare (Figure 1).

**Fig. 2:**
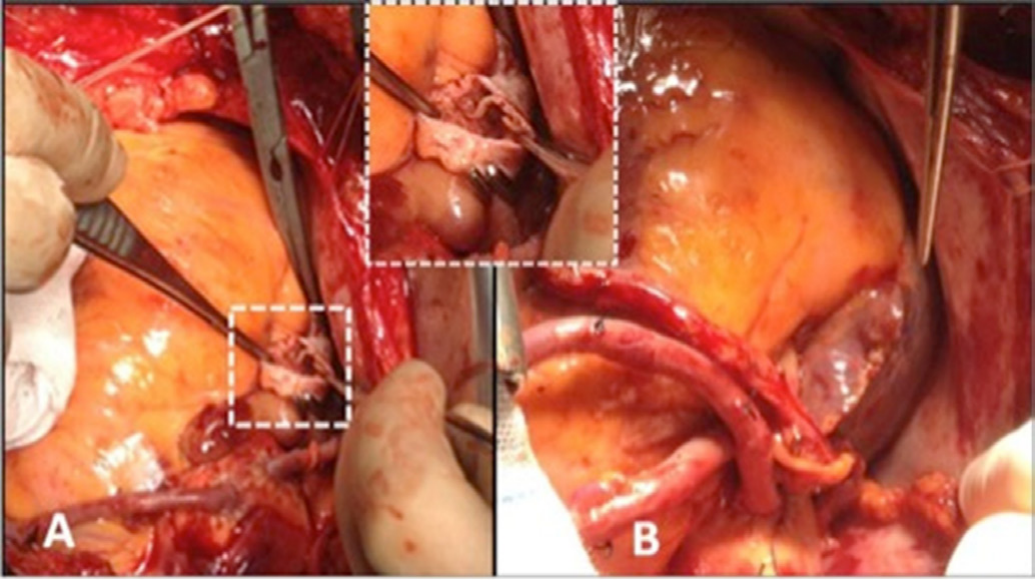
A) Closure of right atrial appendage with side clamp after venous decannulation. B) Primary repair of right atrial appendage.

In the primary repair group (Group B), aortic purse was placed with 2/0 Ethibond suture, and venous purse was placed with 4/0 Prolene™ at the stage of attachment to the heart-lung machine. Following the placement of the aortic cannula, the two-stage venous cannula was inserted into the RA appendage and tightened with the help of purse and snare. After venous decannulation, 4/0 Prolene™ was cut and removed at the decannulation stage, and bleeding in the RA appendage was controlled with the help of a side clamp. Before the aortic cannula was removed, the RA appendage was primarily repaired with 6/0 Prolene™ (Figure 2).

All patients were followed up in the postoperative intensive care unit for one day, and their drains, arterial cannula, and urethral Foley catheter were removed and taken to the clinic. Whole blood and routine tests were studied on the 1^st^ and 3^rd^ postoperative days. Electrocardiogram (ECG) was taken daily in the morning. Temperature, heart rate, and blood pressure measurements were made with routine two-hour follow-ups. ECG was taken again in patients with suspected rhythm disorders. The central venous catheters of the patients who did not need additional blood and whose last blood sampling was routinely taken on the 3^rd^ postoperative day were removed.

### Statistical Analysis

Descriptive statistics are presented with frequency, percentage, mean, standard deviation (SD) and median, and minimum (min) and maximum (max) values. Power analysis was performed statistically to determine the sample sizes. The minimum number of samples, which was determined by taking the power of the test to 80%, was determined, and the study was started. Fisher’s exact test or Pearson’s chi-square test was used to analyze the relationships between categorical variables. Shapiro-Wilk test was used for normality test. In the analysis of the difference between measurement values of the two groups, the assumption of normality was checked with the Shapiro-Wilk test, the Mann-Whitney U test was used when it did not fit the normal distribution, and the Student’s *t* -test was used when it did. The Friedman test was used to compare ANP/BNP values on the preoperative day and postoperative days 1 and 3. In cases where the difference was significant, the Bonferroni-Dunn procedure was applied in pairwise comparisons. *P* -values < 0.05 were considered statistically significant. Analyzes were made with the IBM Corp. Released 2013, IBM SPSS Statistics for Windows, version 22.0, Armonk, NY: IBM Corp. package program.

## RESULTS

In the ligation (group A) and primary repair (group B) groups, age, weight, body mass index, gender, number of grafts, revision number, hypertension, diabetes mellitus, CRF, chronic obstructive pulmonary disease, and peripheral arterial disease were analyzed. There was no statistically significant difference between the two groups when comparing the descriptive variables (*P* > 0.05) (Table 1).

**Table 1:** Comparison of descriptive variables between the two groups.

Variable		Group A		Group B		*P* -value
		Mean ± SD (n)	Median % (min - max)	Mean ± SD (n)	Median % (min - max)	
Age^1^ (years)		66.7 ± 10.34	66.5 (48 - 84)	61.17 ± 9.35	59.5 (46 - 82)	0.094
Weight^2^ (Kg)		83.5 ± 20.95	76.75 (61 - 140)	76.56 ± 9.9	76 (62.5 - 93)	0.529
BMI^1^		29.47 ± 5.08	8.5 (21.98 - 39.6)	28.42 ± 2.75	28.97 (23.8 - 32.5)	0.429
Gender^3^	Female	3	15.0	4	22.2	0.687
					
	Male	17	85.0	14	77.8	
HT^4^	No	7	35.0	7	38.9	0.804
	Yes	13	65.0	11	61.1	
DM^4^	No	11	55.0	9	50.0	0.758
	Yes	9	45.0	9	50.0	
CKD^4^	No	20	100.0	18	100.0	-
	Yes	0	0.0	0	0.0	
COPD^3^	No	19	95.0	17	94.4	0.999
	Yes	1	5.0	1	5.6	
PAD^3^	No	15	75.0	16	88.9	0.41
	Yes	5	25.0	2	11.1	
					

1 Student’s *t* -test,

2 Mann-Whitney U test,

3 Fisher’s exact test,

4 Pearson’s chi-square test

BMI=body mass index; CKD=chronic kidney disease; COPD=chronic obstructive pulmonary disease; DM=diabetes mellitus; HT=hypertension; PAD=peripheral arterial disease; SD=standard deviation

In the transthoracic echocardiographic (Vivid 5, General Electrics, United States of America) measurements of the two groups, the differences between preoperative EF, postoperative EF, preoperative left atrial diameter, preoperative RA diameter, and postoperative 1^st^ day RA values were investigated. No statistically significant difference was found according to the difference tests between the two independent groups ( *P* > 0.05).

The differences between the two groups’ intraoperative CCT, total bypass time (TBT), central venous pressure, and pulmonary artery systolic pressure values were investigated. No statistically significant difference was found according to the two independent difference tests ( *P* > 0.05). The differences of the two groups according to the biochemical parameters obtained on the preoperative and 1st and 3rd postoperative days were evaluated. According to the two independent group difference tests, no difference was observed in sodium, potassium, hematocrit, and hemoglobin measurements ( *P* > 0.05).

When all patients were evaluated, POAF was observed at a rate of 15.78%. It was observed in 30% of the patients in group A, and in none of the patients in group B. POAF rate in group A was found to be statistically significantly higher than in group B ( *P* < 0.05) (Table 2). In group A, AF was observed in one of six patients on day zero, three on the 1^st^ postoperative day, and two on the 2^nd^ postoperative day. The mean AF duration was 66.67 minutes (SD: 45.46 min), and the median was 60 minutes (min: 10 - max: 120).

**Table 2:** Comparison of postoperative atrial fibrillation data between the two groups.

		Group A		Group B		*P* -value
		n	%	n	%	
POAF	No	14	70.0	18	100.0	0.021^*^
	Yes	6	30.0	0	0.0	
Day of AF	0	1	16.7	0	0.0	
	1	3	50.0	0	0.0	
	2	2	33.3	0	0.0	
		Mean ± SD		Median (min - max)		
AF duration (min)		66.67 ± 45.46		60 (10 - 120)		

^^*^^ *P* < 0.05; Fisher’s exact test

AF=atrial fibrillation; POAF=postoperative atrial fibrillation; SD=standard deviation

When ANP/BNP values were compared between the two groups on the preoperative and 1^st^ and 3^rd^ postoperative days, no statistically significant difference was found in any measurement ( *P* > 0.05) (Table 3).

**Table 3:** Comparison of ANP/BNP values on preoperative and 1st and 3rd postoperative days between the two groups.

Test	Group	N	Mean	SD	Median	Min	Max	*P* -value
ANP0^*^	A	20	1146.16	656.47	936.13	657.08	3406.26	0.52
	B	18	6876.67	15580.45	879.26	226.59	50000	
ANP1^*^	A	20	1920.53	1788.56	1289.5	731.58	8149.89	0.29
	B	18	5358.07	11460.79	1728.5	525.40	48000	
ANP3^*^	A	20	1428.56	688.09	1177.2	755.65	3431.74	0.15
	B	18	2899.07	3436.29	1383.8	492.45	13195.2	
BNP0^*^	A	20	43.53	28.30	33.66	17.61	119.60	0.79
	B	18	57.08	60.29	30.76	7.27	193.98	
BNP1^*^	A	20	65.95	40.95	57.69	22.6	162.21	0.43
	B	18	82.13	54.46	57.38	9.89	189.26	
BNP3^*^	A	20	53.80	31.11	45.70	18.30	132.90	0.36
	B	18	73.74	55.30	52.84	15.06	185.15	

* Mann-Whitney U Test, 0=preoperative day, 1=postoperative 1^st^ day, 3=postoperative third day

ANP=atrial natriuretic peptide; BNP=brain natriuretic peptide; SD=standard deviation

## DISCUSSION

The great increase in the incidence of ischemic heart disease in recent years has also increased the need for coronary revascularization. The purposes of CABG, which is widely performed all over the world, are to improve the patient’s quality of life and to provide a long lifespan. Although the development of operative techniques, myocardial protection, and the increase in the quality of postoperative care have greatly reduced the mortality in CABG patients, the morbidity is still high. RA fibrosis and released mediators during venous cannulation and decannulation stage cause an inhomogeneous distribution in diastolic depolarization potentials, refractory periods, and conduction rates in the atrial muscle due to topical cooling^[7]^. As a result, various arrhythmias may occur. Also, the majority of natriuretic peptides originate from the atria, particularly the atrial appendage. In the literature, there are studies with different results showing how their levels are affected in patients undergoing coronary bypass^[8]^. Tension of the atria rapidly leads to ANP release. The secreted ANP/BNP ratio is the same as the storage ratio in mature atrial myocyte granules. It is not fully understood how the mechanical force generated by wall tension is translated into a biochemical response such as secretion^[9]^.

Natriuretic peptides have been frequently researched in recent years that especially ANP and BNP levels may cause or be a precursor of AF^[10]^. Supraventricular arrhythmias occurring after CABG are among the causes of morbidity in the early postoperative period. POAF is the most common supraventricular arrhythmia^[11]^. Reactive changes that occur after atrial ischemia cause local conduction blocks that lead to re-entries in the atrial tissue, and this lays the groundwork for the development of POAF.

POAF is a common complication seen in approximately 20% to 40% of patients after isolated coronary bypass surgery. Creswell et al.^[12]^ evaluated 4507 patients who underwent isolated coronary bypass, and the incidence of POAF was 31.9%. Canbaz et al.^[13]^ reported 18.9% in their study. In our study, it was evaluated as 15.78% in the total number of patients, 30% in the ligated group, and 0% in the primary repair group. Fuller et al.^[14]^, in a study of 1666 patients who applied isolated coronary bypass, found that the development of POAF was most common on the 2^nd^ postoperative day. Canbaz et al.^[13]^ reported that they detected POAF at the earliest in the 3^rd^ hour and at the latest in the 196^th^ hour, and that they saw it at an average of 66^th^ hour (2.75^th^ day). In our study, it was most frequently seen on the 1^st^ postoperative day.

It is important to develop optimal strategies for the prevention of POAF due to complications such as stroke, heart failure, heart attack, thromboembolism, bleeding due to anticoagulation, and readmission to hospital. In almost all of the literature for POAF developing after CABG, advanced age was recognized as the most influential independent risk factor. In the study Leitch et al.^[15]^ conducted in 5807 patients with preoperative sinus rhythm who underwent isolated coronary bypass, 3.7% of those who developed POAF were 40 years old or younger, 27.7% were 70 years old and older, and advanced age as a risk factor was found to be statistically significant for POAF. Zaman et al.^[16]^ published 358 coronary bypasses in the study they applied and reported the mean age of 65.9 years in 92 patients with POAF. Canbaz et al.^[13]^ reported the mean age as 56.1 ± 9.2 years in the sinus group and 62.9 ± 6.4 years in the POAF group ( *P* < 0.05). In our study, the mean age of the patients was 61.17 ± 9.35 years.

While male gender is accepted as an independent risk factor in some studies, there are studies that state the opposite. In the study that Borzak et al.^[17]^ conducted on 436 isolated coronary bypasses, 101 (23%) POAF were seen, and it was stated that 72% of the sinus group and POAF group were male; they reported that gender was not a determining risk factor for the development of POAF. In our study, no significant difference was observed in terms of gender-POAF relationship in both groups ( *P* > 0.05).

The number of distal anastomoses is important in terms of showing the severity of coronary artery disease. Since it may be an indicator of myocardial dysfunction, it may also be an effective factor in the development of POAF. Rubin et al.^[18]^ stated that the amount of coronary artery lesions is not effective in the formation of POAF. In addition, Roffman et al.^[19]^ stated that the frequency of POAF increases as the number of grafts placed increases, and therefore the amount of coronary lesions may be important. In our study, there was no significant difference between the numbers of distal anastomoses in groups with and without POAF ( *P* > 0.05).

There are studies indicating that prolonged CCT is an independent risk factor for the development of POAF, since it also means prolonged atrial ischemia. Canbaz et al.^[13]^ stated that the CCT was 46.4 ± 19 minutes in the sinus rhythm group and 49.1 ± 18 minutes in the POAF group; no statistically significant difference was found between the two groups. In our study, no significant difference was found between the ligation group with POAF and the primary repair group without POAF in terms of TBT and CCT ( *P* > 0.05).

In their study, Omari et al.^[20]^ performed 23 elective coronary bypasses and investigated serum ANP level and postoperative urine output by dividing the patients into two groups as those with venous cannulation with and without preservation of RA appendage. It has been reported that the serum ANP level was significantly higher in patients whose RA appendage was preserved, and their urine output was significantly higher in the postoperative period compared to the other group. Morimoto et al.^[21]^, in the study in which they performed cardiac surgery and examined perioperative ANP and BNP values, observed a significant increase in BNP values in all patients in the postoperative acute period. He evaluated that this increase was due to ischemia secondary to the operation. In 22 of 42 patients who underwent Maze procedure, Yoshihara et al.^[22]^ investigated its effect on plasma ANP level by protecting the RA appendage. They reported that plasma ANP levels were significantly higher than in the other group in the postoperative period and renal fluid excretion was better. In our study, primary repair was performed after venous decannulation, considering the preservation of the RA appendage; there was no significant difference in ANP/BNP levels between groups ( *P* > 0.05).

In our study, RA anatomy was repaired with primary repair, following venous cannulation and decannulation from the appendix, with the aim of minimizing the impact of coronary bypass operations; we tried not to affect the natriuretic peptide cycle released from atrium and to optimally measure its levels in the postoperative period. We did not encounter the primary repair method as a surgical technique in our literature review. At the same time, although there are many studies stating that natriuretic peptides and atrial ischemia are risk factors for POAF, we think that this study, in which we reduced RA ischemia with surgical technique, measured natriuretic peptide, and investigated its relationship with POAF, will contribute to the literature.

### Limitations

This study has several limitations. The sample size was relatively small, which may affect the generalizability of the results. The follow-up period was limited to the early postoperative days, preventing evaluation of long-term outcomes. Additionally, other factors influencing POAF, such as genetic predisposition or detailed intraoperative variables, were not assessed.

## CONCLUSION

As a result, while the number of POAFs developed in the ligation group was statistically significant compared to the primary repair group, there was no statistically significant difference between the two groups in terms of ANP/BNP measurements. POAF was not observed in the primary repair group, in which we surgically reconstructed the RA appendage.

For this reason, preserving the anatomical integrity of the RA appendage is important for POAF. We evaluate that the primary repair method can be effective for the prevention of POAF development.
